# Mass calibrants for positive chemical ionization-high resolution mass spectrometry (CI-HRMS) for the identification of unknown compounds using accurate mass measurements†

**DOI:** 10.1039/d3ra01977b

**Published:** 2023-05-09

**Authors:** Bilal Nehmeh, Fatima Haydous, Elias Akoury

**Affiliations:** a Department of Natural Sciences, School of Arts and Sciences, Lebanese American University Beirut 1102-2801 Lebanon elias.akoury@lau.edu.lb

## Abstract

Gas Chromatography-Electron Ionization-Mass Spectrometry (GC-EI-MS) is still the most routinely performed method for metabolite profiling as compared to other hyphenated techniques. But when it comes to identification of unknown compounds, information on the molecular weight is not readily available because the molecular ion is not always found with electron ionization (EI). Thus, the use of chemical ionization (CI) is envisaged that commonly produces the molecular ion; in combination with accurate mass measurement, this technique would further allow for calculation of sum formulas of those compounds. However, for proper accuracy of analysis, a mass calibrant is needed. We set out to find a commercially available reference material with mass peaks that would qualify the substance as mass calibrant under CI conditions. Six commercially available mass calibrants, FC 43, PFK, Ultramark 1621, Ultramark 3200F, Triton X-100, and PEG 1000, were tested under CI conditions to understand their fragmentation behavior. Our findings indicate that Ultramark 1621 and PFK best fit the expectations of a mass calibrant for HRMS analysis whereby PFK provided a fragmentation pattern similar to EI outcomes thus enabling the use of mass reference tables commonly provided within commercial mass spectrometers. On the other hand, Ultramark 1621 is a mixture of fluorinated phosphazines that shows stable fragment intensities.

## Abstract 2

Gas Chromatography-Electron Ionization-Mass Spectrometry (GC-EI-MS) is still the most routinely performed method for metabolite profiling as compared to other hyphenated techniques.

## Graphical abstract

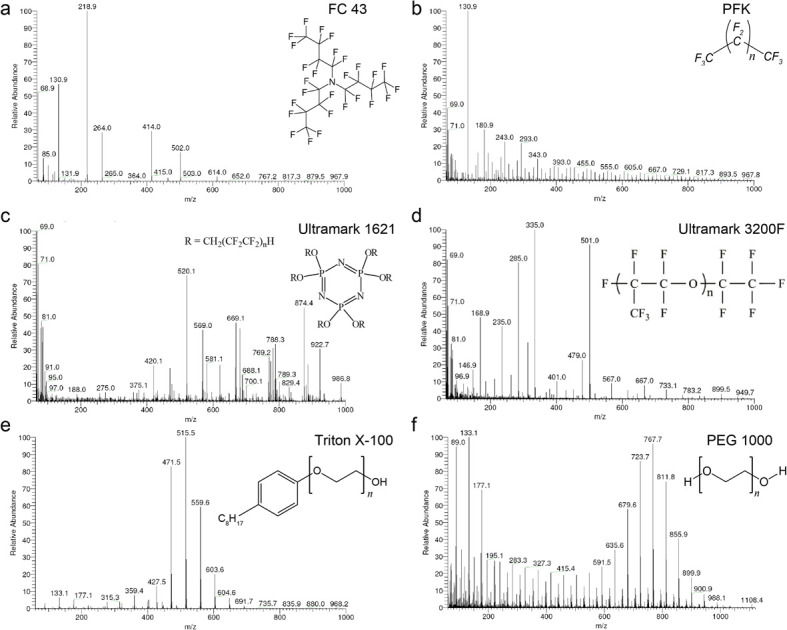

## Introduction

Mass spectrometry (MS) is an indispensable analytical technique employed in various disciplines such as chemistry, biochemistry, physics, pharmacy and medicine.^1,2^ It analyzes sequence biomolecules and combinatorial databases, to explore single cells, to assist with structure elucidation of unknown compounds and to inspect quality of drugs and polymers.^3^ An increasing number of unknown compounds in biological and macromolecular systems possess challenges in the elucidation of their structures; thereby compelling an emergent necessity for high mass, high sensitivity MS.^4,5^ Accurate mass spectrometry enables calculation of theoretical sum formulas for those compounds on the basis of their accurate masses. The sum formula is valuable information when elucidating the nature and structure of a compound.

In the field of metabolite/proteomics profiling, identification of molecules in biological matrices is performed with GCMS or LCMS by means of the features of standardized retention time indices and specific fragmentation after EI ionization.^6–9^ However, when it comes to identification of unknown components in a complex mixture, high-energy fragmentation during EI might be disadvantageous, since a molecular ion is often not produced and information on its molecular weight is not available.^10^ With unknown compounds, it would be difficult to decide about whether the highest *m*/*z* in the EI spectrum is the molecular ion or not. Therefore, during the identification of unknown compounds within a GCMS chromatogram of any complex mixture, the use of chemical ionization (CI) is envisaged as it commonly provides the molecular ion of small molecular weight compounds.^11^ This technique is a soft ionization method that allows the calculation of sum formulas for unknown compounds in combination with accurate mass measurement. CI is correlated with low energy ionization by positive or negative modes under a reagent gas (typically methane, isobutane, ammonia and hydrogen) and results in higher abundant molecular ions. Even though CI provides information about molecular weight for unknown species, the sample must be volatile which could hinder thermally unstable molecules.^12,13^

High resolution (HR) enables the determination of unknown compounds by identifying the elementary composition molecules or fragment ions.^14^ The simultaneous acquisition of reference and sample ions allows the precise calculation of their masses. For instance, HR measurements deliver high selectivity when applied on isobaric compounds that possess the same nominal mass but different accurate masses.^15^ In contrast to low resolution (LR) where only combined measurements and no specific quantification are possible, HR allows individual detection and a separate quantification after elimination of the chemical interference of the same nominal mass but different accurate mass.^16^ This increases substantially the signal-to-noise ratio but decreases sensitivity. In HRMS applications, a mass calibrant is required to adjust the accuracy of the instrument and hence is ideally a compound that produces ions covering the whole desired mass range with sufficiently small mass differences between one ionic species and the next.^17^ The analyte and calibrant should coexist in the ion source simultaneously where they are ionized concurrently to obtain full scan HR accurate mass data with sufficiently resolved peaks. A good calibrant displays a fragmentation pattern distributed over a large *m*/*z* region with a homogenous signal intensity which is well resolved from the analyte peaks. Notably, the ppm error in the mass assignment is proportional to the square of the mass difference between the calibration ions.^17,18^

An appropriate calibrant contains as few hetero atoms and isotopes as possible to facilitate the assignment of reference masses and minimize the occurrence of unresolved multiplets within the reference spectrum. An approximate upper mass limit should assist in the selection of the appropriate reference. A number of reference materials have been identified as potential calibrants for EI and CI-HRMS such as pefluorotributylamine (PFTBA or FC-43), perfluoro-5,8-dimethyl-3,6,9-trioxidodecane (PFDTD), perfluoro kerosene (PFK),^19^ Ultramark 1621/3200F,^20^ and polyethylene glycols (PEGs).^21^ PFTBA and PFDTD are both used to tune commercial spectrometers as they represent masses that are well separated and have zero defects. Although these calibrants offer advantages in negative ion mass calibration, however, both have low vapor pressures and no significant ions below 302 amu. PFK is a viscous molecule widely used for mass calibration under EI conditions but has a low ionization efficiency in CI mode. PFK is usually introduced into the ion source through the heated inlet and a dominant series of ions is produced corresponding to C_*n*_F_2*n*−1_, with other minor series of C_*n*_F_2*n*−3_, C_*n*_F_2*n*−5_ and C_*n*_F_2*n*−7_.^22^ Equally important, Ultramark is a mixture of fluorinated phosphazines applied in the calibration of various HRMS techniques. PFK calibrant comprises a series of intense peaks ranging from 700 to 1900 u at a consistent mass interval of less than 100 u. On the other hand, a higher range can be achieved by the use of Ultramark 3200F, a mixture of tris (perfluoro alkyl ethyl) silyl alkyl amines.^19^ Another recognized calibrant is the family of PEGs with the chemical composition (C_2_H_4_O)_*n*_·H_2_O and are most widely used in positive ion Fast Atom Bombardment (FAB)-HRMS.^23^ The mass spectrum of PEG displays a sequence of intense peaks with an interval of 44 u. interestingly, Triton X-100 is a particular PEG comprising a 4-(1,1,3,3-tetramethylbutyl)phenyl end chain functionality and is widely used as a calibrant for positive-ion ammonia CI-HRMS and positive-ion thermospray mass spectrometry.^24^ Triton X-100 is introduced to a crucible on the direct insertion probe due to its high viscosity and insufficient volatility. The mass spectrum of Triton X-100 displays an envelope of ions centered at *m*/*z* 500–600 and are evenly spaced by 44 u.^24,25^Table 1 represents the major physical–chemical properties of the six calibrants that are center to this study.

**Table 1:** Physical–chemical properties of the six calibrants FC 43, PFK, Ultramark 1621, Ultramark 3200F, Triton X-100, and PEG 1000

Calibrant	FC 34	PFK	Ultramark 1621
Nomenclature	Perfluoro tributylamine	Perfluoro kerosene	Perfluoro alkyl phosphazene
Molecular formula	C_12_F_27_N	C_*n*_F_2*n*+2_	C_42_H_18_F_72_N_3_O_6_P_3_
Structural formula	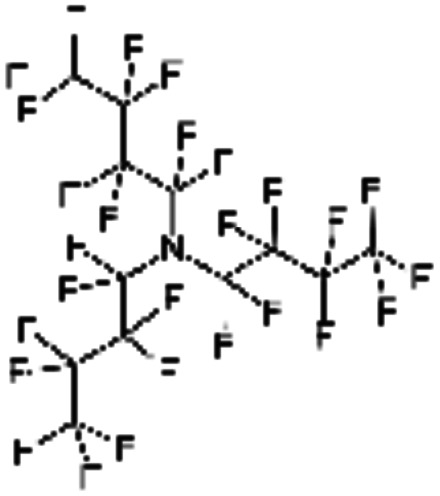	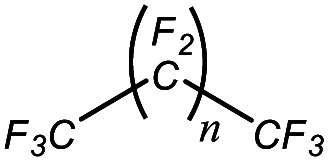	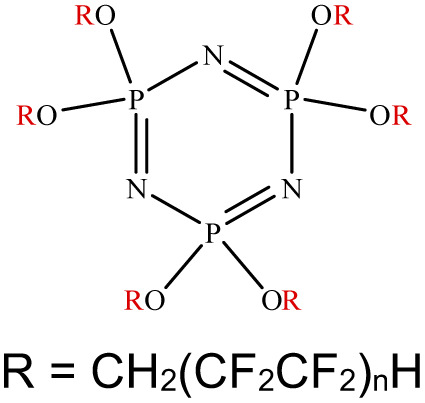
Molecular weight	671.09 g mol^−1^	Low boiling: *n* = 10 600 g mol^−1^	2120.41 g mol^−1^
		High boiling: *n* = 17 800–900 g mol^−1^	
Calibrant	Ultramark 3200F	Triton X-100	PEG 1000
Nomenclature	Tris (perfluoro alkylethyl) silyl alkyl amine	4-(1,1,3,3-tetra methylbutyl) phenyl-polyethylene glycol	Polyethylene glycol
Molecular formula	R_3_Si(CH_2_)_3_N(R)(CH_2_)_3_SiR_3_	C_34_H_62_O_11_	H(OCH_2_CH_2_)_*n*_OH
Structural formula	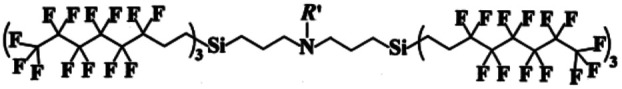	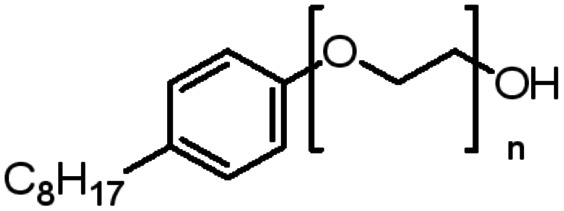	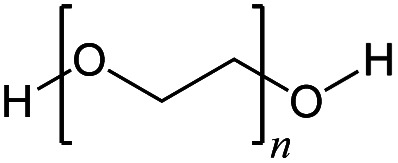
Molecular weight	1000–3000 g mol^−1^	646.86 g mol^−1^	950–1050 g mol^−1^

Mixing of calibrants is a commonly practiced technique for achieving an appropriate fragmentation coverage over a wide *m*/*z* range.^26^ For instance, when a mixture of calibrants was combined with a glycerol matrix, the signal stability in a FAB MS application was dramatically enhanced, sample sputtering was reduced and source contamination minimized.^27^ The combination of PFK and Ultramark 1621 is equally important where PFK is beneficial for calibrating positive mode CI MS/MS up to *m*/*z* 1200 while Ultramark 1621 is more practical in applications above *m*/*z* 1000.^28^ this combination extends the mass range up to *m*/*z* 2000. Similarly, Fomblin is readily used in negative ion mode CI MS/MS applications up to *m*/*z* 1200;^28^ which is also extended to above 2000 when combined with Ultramark 1621.^17^

In our current study, we investigated six commercially available reference materials with known fragmentation peaks arising under CI conditions that would qualify these candidates as mass calibrants in accurate mass measurements. Two promising calibrants were intensively tested to achieve mass calibration on an HRMS double sector mass spectrometer under isobutane and methane CI conditions.

## Materials and methods

### Chemicals and reagents

Perfluoro tributylamine (FC 43), Perfluoro kerosene (PFK), perfluoro alkyl phosphazene (Ultramark 1621), tris(perfluoro alkylethyl) silyl alkylamine (Ultramark 3200F), 4-(1,1,3,3-tetramethylbutyl)phenyl-polyethylene glycol (Triton X-100), and polyethylene glycol (PEG 1000), ribose (C_5_H_10_O_5_; 150.0528 g mol^−1^), kaempferol (C_15_H_10_O_6_; 286.0477 g mol^−1^), 1,3,5-triphenyl benzene (C_24_H_18_; 306.1408 g mol^−1^) and pyrene (C_16_H_10_; 202.0783 g mol^−1^) were all purchased from Sigma Aldrich Chemie GmbH (Schnelldorf, Germany).

### Low resolution mass spectrometry

All experiments were carried out on a Thermo Finnigan MAT 95 XP double focusing sector field mass spectrometer (Thermo Fisher Scientific GmbH, Bremen, Germany) using Xcalibur software. Calibrants were first evaluated in low resolution mode and depending on the nature of the reactant gas, the ion source was tuned at *m*/*z* 57 and 17 for isobutane and methane, respectively. The behavior of the six calibrants was evaluated during CI for different ionization parameters. The six calibrants (10 to 30 μg l^−1^) were evaluated independently with isobutane and methane reactant gases after adapting the temperature to 200 °C, the plasma pressure at 2.0 × 10 ^−4^ mbar. The current filament was adjusted at 0.2 mA for isobutane and 0.1 mA for methane and the energy source of the electrons at 150 eV for isobutane and 100 eV for methane, respectively. FC 43 and PFK were introduced directly into the ion source *via* the reference gas inlet whereas the other four were introduced with the direct insertion probe with probe temperature of 100 °C. The rate of sample evaporation was allowed to stabilize thus enabling reproducible measurements. Ultramark 1621 and PFK were further investigated with isobutane to probe the influence of different parameters and find the optimal conditions such as variation of the reagent gas pressure (10^−5^ to 10^−4^ mbar), filament current (0.1 to 0.2 mA), source temperature (150 to 200 °C) and electron energy (150 to 200 eV). Ultramark 1621 was further tested with methane at 200 °C under the same conditions while changing furthermore the electron energy (100 to 130 eV) as recommended for this reagent gas. Ultramark 1621 main fragments (*m*/*z* 922, 1022 and others) were further investigated with Collision Induced Dissociation Mode (CID) using Esquire 3000Plus electron spray ionization (ESI) MS ion trap-type instrument (Bruker Daltonics GmbH, Bremen, Germany) to decipher the fragmentation processes.

### High resolution mass spectrometry

High resolution provides the basis to reach high mass accuracy. High resolution is largely influenced by adjustment of the entrance and exit slits, the beam rotation and the focus quad lens. The sweep width was set at 0.05% and the instrument was calibrated to a resolution of 8000 (10% valley definition), after adjusting the entrance and exit slits to 190 and 150, respectively, at a magnet scan rate of 20 s dec^−1^. High resolution is achieved by providing high vacuum and adjusting parameters for the width of the entrance and exit slits, and ion source tune parameter. The pressure reached around 10^−9^ mbar, to avoid disturbance by ion scattering directly influencing reduction of resolution, sensitivity, and deterioration of peak shape. The pressure inside the ion source region reached 10^−4^ mbar for CI and 10^−7^ mbar for EI. Four standard compounds (10 μg l^−1^) selected with respect to variation of molecular weight and polarity (ribose, kaempferol, 1,3,5-triphenyl benzene and pyrene) were measured in two independent experiments under high resolution conditions. PFK was used as internal and external calibrant, respectively and the mass calibration was optimized within the data system to assign digital-to-analog conversion values to mass spectral peaks of known *m*/*z* values. Magnetic and electric scans were calibrated to obtain a correct mass indication. The stability and linearity of the electric scan insured that the accelerating voltage is very suitable for high resolution measurements of limited mass ranges for accurate mass determination which was performed with *R* > 5000. The six potential calibrants (FC 43, PFK, Ultramark 1621, Ultramark 3200F, Triton X-100 and PEG 1000) were subsequently investigated to decipher their fragmentation behavior for further in-depth analysis towards CI-HRMS. Isobutane and methane were selected as reactant gases in chemical ionization. Ammonia was tested but discarded as a reactant gas due to its high proton affinity.

## Results and discussion

### Selection of proper calibrants

For the selection of a proper calibrant, the data of the low resolution measurements was subjected to numerical analysis including the analysis of variance ANOVA.^29,30^ The use of 3 thresholds of relative signal intensity (0.0001, 0.001 and 0.01) showed no significant differences and hence the highest noise of 0.01 was selected *i.e.* the minimum relative signal intensity of a calibrant peak to be used for mass calibration. Each of the calibrants was evaluated as total data and as “bin” data using the relative intensities obtained. The total data includes the entire values of relative signal intensity where average (Avg), standard deviation (SD), relative standard deviation (RSD), and the difference between the average and median of the relative signal intensities have all been estimated. RSD is calculated from the standard deviation and the average. The bin data was obtained after collecting the relative intensities in sets of 50 amu *m*/*z* windows (denominated as “bin”) over the range 60 to 1000. 50 amu was chosen with respect to the fact that with E-scans the appropriate mass range is 50 amu. The ultimate calibrant would be the one with the highest number of mass peaks per bin for the highest noise threshold, lowest RSD of relative signal intensities and lowest difference between the average and the median of the relative signal intensities within a bin. Highest count per bin provides an idea of even distribution of the fragments, lower RSD paired with the lowest difference between the average and the median indicates even distributed ion intensities.

**Fig. 1:**
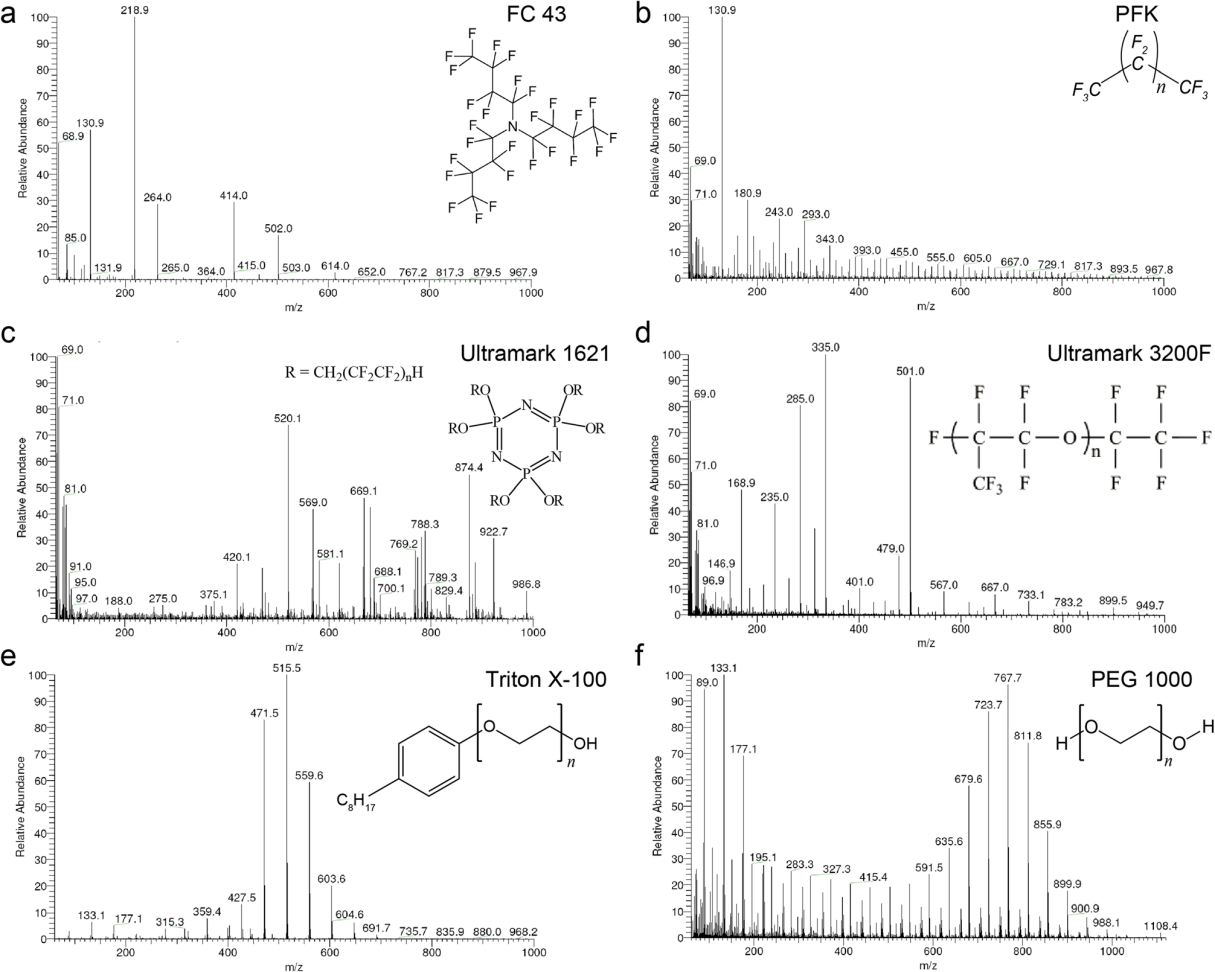
Positive CI Mass Spectra of six calibrants with Isobutane reactant gas. Low resolution mass spectra of (a) FC 43, (b) PFK, (c) Ultramark 1621, (d) Ultramark 3200F, (e) Triton X-100, and (f) PEG 1000 with isobutane reactant gas acquired on Thermo Finnigan MAT 95 XP double focusing sector field spectrometer operated at 200 °C, plasma pressure of 2.0 × 10^−4^ mbar, current filament of 0.2 mA and electrons energy source of 150 eV.

To investigate potential mass calibrants in accurate mass measurements, we first acquired mass spectra of the six calibrants in positive CI mode under isobutane (Fig. 1) and methane (ESI Fig. 1†) reactant gases. The overview of the six calibrants with methane looks similar to their behavior with isobutane. However, methane allows for broader fragmentation patterns. FC 43 shows poor fragment distribution even at low pressure and is not a promising calibrant for high CI-HRMS. On the other hand, PFK showed intense fragments upon introduction into the ion source. However, the intensity diminishes dramatically in short time preventing stable analytical conditions. Moreover, the use of PFK showed severe difficulties with pressure adjustment. For the remaining four calibrants, the probe temperature was an additional parameter to monitor. Being a composition of low and high boiling compounds, Ultramark 1621 fragments sufficiently at probe temperatures between 80 and 120 °C. At higher pressure, the fragments corresponding to the high *m*/*z* values are more intense however the overall intensities diminish at lower pressure. Remarkably, Ultramark 3200F shows proper fragmentation pattern at probe temperature of 170 °C but with a low overall intensity. The candidate Triton X-100 fragments poorly even at a probe temperature of 160 °C. Triton X-100 is rather volatile and its incorporation with GCMS would be problematic since a constant evaporation rate is required over the whole run to ensure stable ion intensities. On the other hand, the spectrum of PEG 1000 at a probe temperature of 180 °C represents a homogenous fragmentation pattern over the whole range of interest with the clear series of Δ*m* = 44 amu. At higher probe temperatures, an increase in the intensities of the fragments in the high range is obvious, eventually caused by higher evaporation rates of the higher boiling components of the calibrant.

The statistical analysis of the calibrants ionization is represented in Table 2. Our analysis indicates that PFK, PEG 1000, and, particularly, Ultramark 1621 showed the preeminent results in terms of highest overall number of mass peaks in the bins, lowest RSD and lowest difference between average and median with respect to the relative signal intensities. However, the overall elemental composition of PEG 1000 is potentially problematic since it is expected to interfere with many organic analytes. On a side note, FC 43 was not considered further as a component in a potential mixture with the other calibrants since it does not fragment properly at higher pressure and does not show sufficient fragment mass peaks in the lower mass range. Among the tested compounds, only PFK and Ultramark 1621 provided the desired mass deficiency to avoid the overlap with typical organic formulas and hence were selected as the two most promising candidates for CI-HRMS analysis of unknown organic compounds.

**Table 2:** Statistical analysis of the calibrants ionization with isobutane and methane^a^

	FC 43	PFK	Ultramark 1621	Ultramark 3200F	Triton X-100	PEG 1000
**(a) Ionization with isobutane at 150 °C**						
Intensity (a.u.)	2 802 000	200 000	3 700 000	1 200 000	900 000	543 000
Average count (in bin)	7	39	43	26	22	48
Average RSD per bin	223.82	198.68	154.07	213.73	256.26	215.20
Average − Median	2.22	0.76	0.42	1.04	0.63	1.64

**(b) Ionization with methane at 150 °C**						
Intensity (a.u.)	5 304 000	100 000	5 400 000	2 700 000	1 000 000	382 000
Average count (in bin)	11	28	48	26	35	47
Average RSD per bin	179.60	202.76	202.84	319.43	220.58	175.24
Average − Median	0.92	0.27	0.86	1.15	0.72	0.66

a After ionization with (a) isobutane and (b) methane, calibrants were evaluated as total data and as bin data using the relative intensities of relative signal intensity (average = Avg, standard deviation = SD, relative standard deviation = RSD). The bin data was obtained after collecting the relative intensities in sets of 50 amu *m*/*z* windows denominated as bin over the range 60 to 1000.

### Optimization of conditions for PFK and Ultramark 1621

The PFK spectra acquired in CI-MS with isobutane at 150 °C are reported in ESI Fig. 2† and optimal conditions of important parameters such as source pressure, source temperature, filament current, and electron energy are evaluated *via* statistical analysis (ESI Table 1†). An increase in pressure and accelerating voltage results in a decrease in the average count per bin (the average number of mass calibrant peaks per bin) and in the RSD but with no significant change in the difference between the average and the median. This indicates either a less extent in fragmentation or decreased overall intensity, so that signal intensities of less abundant fragments fall under the detection limit. It is important to note that the filament current had no substantial influence on the three statistical parameters. The two optimal conditions (1 and 5 in ESI Table 1†) serve for CI-HRMS with isobutane as reagent gas and PFK as mass calibrant. The second candidate, Ultramark 1621, was investigated thoroughly where mass spectra were acquired with isobutane at 150 °C (ESI Fig. 3†) and at 200 °C (ESI Fig. 4†). Optimization conditions are represented in ESI Table 2a and b.† The increase in pressure resulted in a decrease of the difference between the average and the median whereas the filament current and electron energy did not impact significantly. The source temperature (200 °C *versus* 150 °C) did not have a noteworthy influence however the highest employed pressure of 2 × 10^4^ mbar did impact the results. Thus, among the tested parameters, only the pressure showed considerable influence on the mass spectra with the correlating parameter being the difference between the mean and median. For comparison, the optimization of Ultramark 1621 at 200 °C using methane was further analyzed (ESI Fig. 5 and ESI Table 2c†). While average count and average RSD do not significantly change in response to variation of the filament current, electron energy, and source pressure, the difference between mean and median is a projecting variable that follows source pressure variation.

To better understand the significance of optimal parameters on the fragmentation of the candidates, the datasets were further analyzed using ANOVA. Besides dependencies between the parameters, ANOVA tests the statistical significance of the influence exhibited by the investigated parameter on the numerical variable acquired.^29,30^ This test simultaneously compares all means and reports whether there is variation in the means across a number of groups. The purpose of ANOVA was to determine whether differences in group means are significantly large after accounting for differences in the variances within groups. It compares differences between group means by decomposing the total variance in the data into within-group variance and between-group variance. If the between-group variance is sufficiently larger than the within-group variance, then the test concludes that there are differences between the means of the groups. By using two-factorial ANOVA with sample replication, the data contained in a spectrum is transformed to a characteristic value, representing the spectrum. We tested sum and average of the relative signal intensities of the fragments belonging to each spectrum obtained using a particular set of experimental parameters. Table 3 reports the ANOVA analysis calculated from the mean value to study the relation of three parameters: intensity (*I*), pressure (*P*) and accelerating voltage (*E*). Notably, *F*, *P*_value_, and *F*_critical_ values are three outcomes of the analysis where *F* is the ratio of variability between groups after variability treatment within groups due to random error. *P*_value_ is the probability of getting a small *F* value; and needs to be smaller than 0.05 to suggest a significant influence. When the value of *F* is significantly larger than the value of *F*_critical_ then all the parameters are expected to be related. The outcome of the analysis (Table 3) indicates that there exists a significant influence of pressure *P* on the fragmentation pattern without any interactions between *E*, *I* and *P*. ANOVA analysis informs about the dependencies of the information to the parameters and concludes the most crucial parameter on the fragmentation pattern.

**Table 3:** ANOVA Outcome for Ultramark 1621 in methane at 200 °C ANOVA analysis calculated from the mean value relates the three parameters intensity (*I*), pressure (*P*) and accelerating voltage (*E*)^a^^,^^b^

	Pressure			Source of variation	SS	df	ms	*F*	*P*_value_	*F*_critical_
	1.0 × 10^−5^	1.0 × 10^−4^	2.0 × 10^−4^							
***E* = 100**										
*I* = 0.1	944.6384	374.9905	415.5355	between groups	516 810.4	2	258 405.2	73.87065	0.013356	19.00003
*I* = 0.2	1129.7551	413.9994	457.6039	within groups	6996.154	2	3498.077			

***E* = 130**										
*I* = 0.1	1327.68	399.4625	496.1122	between groups	642581.2	2	321 290.6	13.04184	0.071216	19.00003
*I* = 0.2	833.0489	331.0528	322.1264	within groups	49270.74	2	24 635.37			

***I* = 0.1**										
*E* = 100	944.6384	374.9905	415.5355	between groups	1 184 435.281	5	236 887.056	8.865644	0.015878	5.050338
*E* = 130	1327.711	399.4855	496.1382	within groups	133 598.326	5	26 719.665			

***I* = 0.2**										
*E* = 100	1129.718	413.9764	457.5809	between groups	1 155 966.39	2	577 983.19	21.85991	0.001757	5.143249
*E* = 130	833.0489	331.0528	322.1264	within groups	158 642.008	6	26 440.334			

a The analysis outcomes are represented by *F* (the ratio of variability between groups after variability treatment within groups due to random error) *P*_value_ (the probability of getting a small *F* value *i.e.* <0.05 for significant influence) *F*_critical_ (if *F* > *F*_critical_ then all parameters are related).

b SS = sum of squares; df = degrees of freedom; ms = mean squares.

### HRMS measurements using external and internal calibrant

Mass accuracy highly depends on the scanning method, scan rate, resolving power, peak shapes, S/N ratio and overlap of isotope peaks at same nominal mass.^17^ The mass tolerance for routine applications should be within ±5 ppm independent from the ionization method and instrument.

**Fig. 2:**
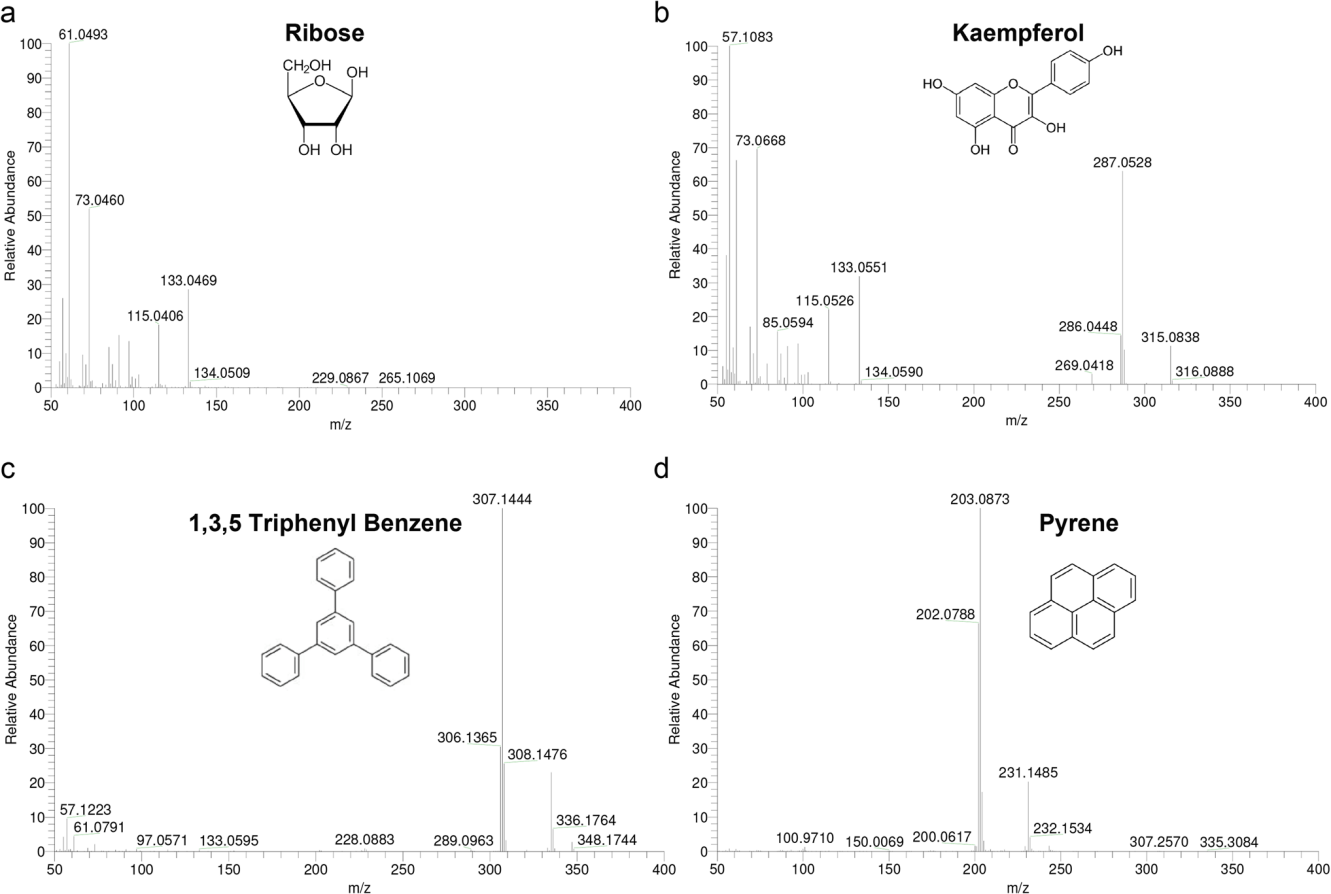
Positive CI HRMS of standard compounds. High resolution mass spectra of (a) ribose (150.0528 g mol^−1^), (b) kaempferol (286.0477 g mol^−1^), (c) 1,3,5-triphenyl benzene (306.1408 g mol^−1^), and (d) pyrene (202.0783 g mol^−1^) with isobutane reactant gas acquired on Thermo Finnigan MAT 95 XP double focusing sector field spectrometer operated at 200 °C, plasma pressure of 2.0 × 10^−4^ mbar, current filament of 0.2 mA, electrons energy source of 150 eV, magnetic scan of 20 s dec^−1^, sweep width of 0.05%, resolution 8000 (10% valley).

A fundamental problem encountered with high resolution CI is a lack of suitable mass calibrants. The use of substituted 1,3,5-triazines as markers and a mixture of polydimethylsiloxanes (PMS) suitable for high resolution CI using methane and isobutane as reagent gases have been reported. The usual mass calibrants PFK, PFTBA, and Fomblin were reported to be unsatisfactory under ammonia CI conditions.^28^ The application of PMS and Triton-x 100 was limited for ammonia CI since the mixture generated primarily M-NH_4_^+^ adduct ions for each oligomer providing a calibration range of 268 < *m*/*z* < 1105. To establish the mass reference table for Ultramark 1621, we measured ESI-MS/MS spectra (data not shown) and assigned the fragments to the proper parent ions using collision-induced dissociation analysis. The molecular formulas of the observed fragments were interpreted as presented in Table 4. In order to facilitate formulation of sum formulas, we then measured four standards (ribose, kaempferol, 1,3,5-triphenyl benzene and pyrene) with CI-HRMS using PFK to check whether the mass accuracy obtained with CI is sufficiently small to use it for mass analysis (Fig. 2). Ribose was excluded due to low intensity of the molecular ion fragment and the masses of the three others were determined using PFK as an external and internal mass calibrant (Table 5). By comparison of the two calibration types, internal calibration shows at least a two-fold better accuracy as expected. However, even with external calibration, results were close to the desired mass tolerance of ±5 ppm and became obvious for higher *m*/*z* values that were tested using Ultramark 1621 (Table 5). The accuracy achieved for Ultramark 1621 fragmentations with external and internal PFK calibration was highly comparable and all calculated tolerances fell in the desired mass range.

**Table 4:** Ultramark 1621 calculated reference table

Ultramark 1621 calculated reference fragments			
Fragment	Exact mass	Fragment	Exact mass
C_42_H_18_F_72_N_3_O_6_P_3_	2120.9259	C_14_H_13_F_20_N_3_O_5_P_3_	775.5893
C_30_H_19_F_48_N_3_O_6_P_3_	1521.9720	C_13_H_16_F_16_N_3_O_5_P_3_	691.0008
C_24_H_19_F_36_N_3_O_6_P_3_	1221.9912	C_12_H_18_F_12_N_3_O_6_P_3_	621.0113
C_22_H_19_F_32_N_3_O_6_P_3_	1121.9976	C_11_H_16_F_12_N_3_O_5_P_3_	591.0008
C_20_H_19_F_28_N_3_O_6_P_3_	1022.0041	C_10_H_12_F_12_N_3_O_4_P_3_	558.2944
C_19_H_19_F_27_N_3_O_6_P_3_	991.0016	C_10_H_17_F_8_N_3_O_6_P_3_	520.0064
C_19_H_18_F_27_N_3_O_6_P_3_	989.9938	C_9_H_15_F_8_N_3_O_5_P_3_	489.3191
C_18_H_19_F_24_N_3_O_6_P_3_	922.0191	C_8_H_12_F_8_N_3_O_5_P_3_	474.2957
C_17_H_16_F_24_N_3_O_5_P_3_	891.0008	C_8_H_12_F_8_N_3_O_4_P_3_	458.3008
C_16_H_13_F_24_N_3_O_5_P_3_	875.5829	C_8_H_17_F_4_N_3_O_6_P_3_	420.0128
C_15_H_16_F_20_N_3_O_5_P_3_	791.0008	C_7_H_15_F_4_N_3_O_5_P_3_	389.2191

CI-HRMS data of standards and Ultramark 1621 with PFK Calibrant

**Table:** 

	External calibrant PFK				Internal calibrant PFK			
	[M + H]^+^	Most abundant peak	External calibration	ppm	[M + H]^+^	Most abundant peak	External calibration	ppm
Kaempferol	C_5_H_11_O_6_	287.0550	287.0528	7.6	C_5_H_11_O_6_	287.0550	287.0547	1
1,3,5-triphenyl benzene	C_24_H_19_	307.1418	307.1444	8.5	C_24_H_19_	307.1418	307.1426	2.6
Pyrene	C_16_H_11_	203.0777	203.0786	4.4	C_16_H_11_	203.0777	203.0772	2.5

**Table:** 

	External calibrant PFK				Internal calibrant PFK			
	Fragment	*m*/*z*	External calibration	ppm	Fragment	*m*/*z*	External calibration	ppm
Ultramark 1621	C_9_H_15_F_8_N_3_O_5_P_3_	489.0019	489.0028	1.8	C_9_H_15_F_8_N_3_O_5_P_3_	489.0019	489.0035	3.3
	C_12_H_18_F_12_N_3_O_6_P_3_	621.0113	621.0142	4.3	C_12_H_18_F_12_N_3_O_6_P_3_	621.0113	621.0153	6.4
	C_18_H_19_F_24_N_3_O_6_P_3_	922.0104	922.0124	2.7	C_18_H_19_F_24_N_3_O_6_P_3_	922.0104	922.0143	4.2

## Conclusion

With the aim of identifying candidates as mass calibrants in positive CI-HRMS for accurate mass measurements, we investigated six commercially available reference materials with known fragmentation peaks under isobutane and methane reactant gases. The experimental parameters of two calibrants, PFK and Ultramark 1621, were further optimized and mass accuracy was evaluated under various conditions. PFK is introduced as a viscous liquid *via* the reference inlet and provides a fragmentation pattern similar to EI, hence enabling the use of readily available mass reference tables for this candidate. On the other hand, Ultramark 1621 is a mixture of fluorinated phosphazenes introduced to the source in a sample cup by the direct insertion probe. Fragment intensity of Ultramark 1621 generally showed better stability, however, the peak pattern obtained was dependent on probe temperature, since different phosphazenes exhibit different vapor pressures. The evaporating low boiling fractions cover the low mass range only while at probe temperatures higher than 100 °C the evaporating high boiling fractions cover the higher mass ranges only.

Our current study has identified Ultramark 1621 and PFK as most prominent calibrants for positive mode CI-HRMS. The ultimate calibrant would provide stable analytical conditions for at least an hour of experiment, *e.g.* to facilitate one GC-CI-HRMS run. Unfortunately, with Ultramark 1621, one needs to consider that the mass range coverage by the calibrant changes by time. With GC, compounds are mainly separated by their boiling points which are in turn proportional to their molecular masses. Ideally, the aim is to cover the whole mass range at a defined probe temperature. Eventually for GC-CI-HRMS, we need to consider a mixture that offers a homogenous fragmentation over the whole mass range of interest for the whole analysis time.

Mass accuracy is highly dependent on many parameters such as resolving power, scan rate, scanning method, S/N ratio of the peaks, peak shapes, and overlap of isotope peaks at same nominal mass, mass difference between adjacent reference peaks as well as others. We expect lower accuracy in CI-HR then in the EI-HR since the resolution is inversely proportional to pressure, though this effect should be mainly restricted to the analyzer. We found that the accuracy of the analysis is absolutely comparable with EI results, so that the mass accuracy should not be critical when determining the exact masses of unknown peaks with CI in routine applications. However, under GC conditions, the mass accuracy needs to be checked, in particular in response to alterations of the scan rate. For structure elucidation, usually an accuracy of less than 5 ppm is required.

## Author contributions

B. Nehmeh and F. Haydous analyzed the data and wrote the manuscript, E. Akoury designed the study, conducted MS experiments, analyzed the data, and wrote the manuscript.

## Conflicts of interest

The authors declare that they have no competing interests.

## Supplementary Material

RA-013-D3RA01977B-s001
