# Supplementary material for: Mass calibrants for positive chemical ionization-high resolution mass spectrometry (CI-HRMS) for the identification of unknown compounds using accurate mass measurements
Source: RSC Adv. 2023 May 9;13(20):14001–9. doi: 10.1039/d3ra01977b (PMC10167574; doi:10.1039/d3ra01977b)
Supplement: RA-013-D3RA01977B-s001 [file RA-013-D3RA01977B-s001.pdf]

Electronic Supplementary Material (ESI) for RSC Advances.
This journal is © The Royal Society of Chemistry 2023

Mass Calibrants for Positive Chemical Ionization-High Resolution Mass Spectrometry (CI-HRMS) for The Identification of Unknown Compounds using Accurate Mass Measurements

Bilal Nehmeh[1*], Fatima Haydous[1*], Elias Akoury[1,2]

[1] Department of Natural Sciences, School of Arts and Sciences, Lebanese American University, Beirut 1102-2801, Lebanon

[2] Correspondence to elias.akoury@lau.edu.lb

* These authors contributed equally

Supplementary Information

Supplementary Writing

The mass accuracy is defined as the difference between measured accurate mass and calculated exact mass; and can be reported as absolute units of u or mmu (millimass unit where 1 mmu is 10^{-3} u), or as relative mass accuracy in ppm. The relative mass accuracy is the absolute mass accuracy divided by the mass determined from m/z. Absolute mass accuracy represents a more meaningful way than the trendier use of ppm. Root mean square, RMS, is calculated and reported together with the absolute mass accuracy.

$$RMS\ Mass\ Accuracy = \sqrt{\frac{\sum(Mass\ Accuracy\ or\ Mass\ difference)^2}{Number\ of\ peaks}}$$

A magnetic sector mass spectrometry allows for an absolute mass accuracy of 2 to 5 mmu[1] in scanning mode over a range of about m/z 50 to 1,500. We consider the following example: at m/z 1,200 an error of 3.5 mmu corresponds to 2.9 ppm, whereas the same error yields 70 ppm at m/z 50 which seems to be unacceptably large. With the use of appropriate internal standards, a mass accuracy of 5 ppm can be attained over a very large mass calibration range between 400 m/z and 2,000 m/z. Below 400 m/z an RMS error of less than 3 mmu can be attained.[2] The most frequently used terms

in MS when discussing fragmentation peak values are exact mass, nominal mass and isotopic mass. The exact mass is defined as the exact mass of the most abundant naturally occurring monoisotopic stable isotope of an element. The nominal mass is defined as the integer mass of the most abundant naturally occurring monoisotopic stable isotope of an element. The isotopic mass is the (exact) mass of an isotope. Mass accuracy ideally is obtained from sufficiently sharp and evenly shaped signals that are well separated from each other. Several definitions of resolution are used in mass spectrometry so it is important to understand the distinctions between these terms. Unit Resolution is the separation of a mass from the next integer mass, such as the separation of a mass 50 from 51 or 1,000 from 1,001. The Resolving power R is the ability of an instrument to perform a separation of neighboring peaks according to equation 2.2 representing the ratio of the mass of interest, m, to the difference between two masses that can be separated, Δm, as defined by the width of a peak at a specific fraction of the peak height or between the two apexes. Resolving power is a performance parameter characterizing the instrument. It is reported by indicating the R value together with the specific m/z value and the method used for Δm determination, either percentage valley or FWHM. Low resolution, LR, is generally used to describe spectra obtained at R between 500 and 2000 whereas high resolution, HR, is for R values larger than 5,000.[1] A resolving power of R 1000 refers to a mass of 1000 Da and Δm of 1.0 Da whereas R of 10000 represents a mass of 1000 Da and Δm of 0.1 Da. For better understanding, we consider the difference between the definition of unit resolution and resolving power as defined by the magnetic sector MS. If we have 5,000 resolving power, we can separate m/z 50.000 from m/z 50.010, or m/z 100.000 from 100.020, or 1,000.000 from 1,000.200, all down to 10% valley between the two peaks. Unit resolution would allow you to distinguish m/z 50 from m/z 51, or m/z 100 from 101, or 1000 from 1001.

The mass spectrometer is a double focusing sector field instrument with reverse Nier – Johnson geometry where the ions traverse the separating magnetic field before entering the electric sector. After passing the electric sector, the ions are converted by the conversion dynode to electrons. The resulting electron current is amplified by the secondary electron multiplier. The maximum magnetic field strength and the maximum accelerating voltage of 5000 volts provide a mass range of 1 to 3500 units; reducing the accelerating voltage can extend this mass range. Thus a mass range of about 17000 units can be obtained when reducing the accelerating voltage to 1000 volts, but

with loss in signal intensity.[3] The reference and reagent gas inlet system is a heated double inlet system. The reference inlet section serves to introduce the reference sample for mass calibration. The reagent gas inlet section serves to introduce the reagent gas for chemical ionization. Viscous and solid analytes are introduced via the direct insertion probe, DIP.

DIP serves to directly evaporate the sample from a crucible into the ionization chamber of the ion source. Two types of crucibles are available, glass crucibles and aluminum crucibles both working at temperatures up to 360°C. Solid crystals of the analyte are introduced into the crucible or 1µl of liquid. High resolution is achieved by providing certain conditions (e.g. high vacuum) and adjusting parameters such as width of the entrance and exit slits, and ion source tune parameter. The vacuum conditions required for MAT 95 XP mass spectrometer regions are different: within the analyzer and ion collector section, the pressure should be as low as possible, usually around 10^{-9} mbar, to avoid disturbance by ion scattering directly influencing reduction of resolution, sensitivity, and deterioration of peak shape. Compared to this, the pressure inside the ion source region can be relatively high, about 10^{-4} mbar for CI and 10^{-7} mbar for EI. Both the entrance (ion source) slit and the exit (collector) slit are the main parameters that define the resolution of the mass spectrometer. Adjustment of resolution is performed by manipulating the entrance and the exit slits since the peak widths and proper shape are dependent on the settings of these two slits.

Supplementary Figures

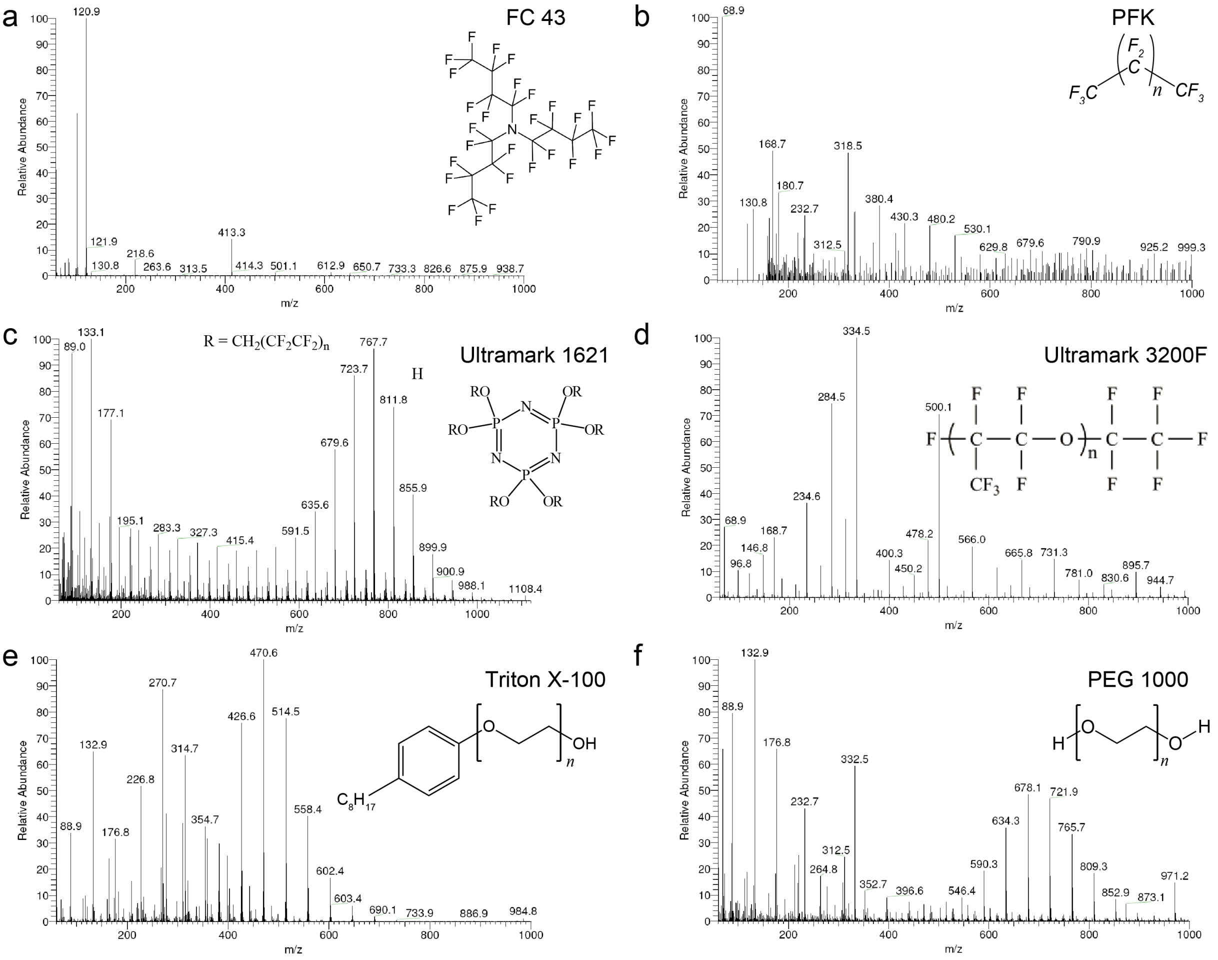

Supplementary Figure 1 – CI Mass Spectra of the six calibrants with methane reactant gas

Low resolution mass spectra of (a) FC 43, (b) PFK, (c) Ultramark 1621, (d) Ultramark 3200F, (e) Triton X-100, and (f) PEG 1000 with methane reactant gas acquired on Thermo Finnigan MAT 95 XP double focusing sector field spectrometer operated at 200^{0}C, plasma pressure of 2.0 x 10^{-4} mbar, current filament of 0.1 mA and electrons energy source of 100 eV.

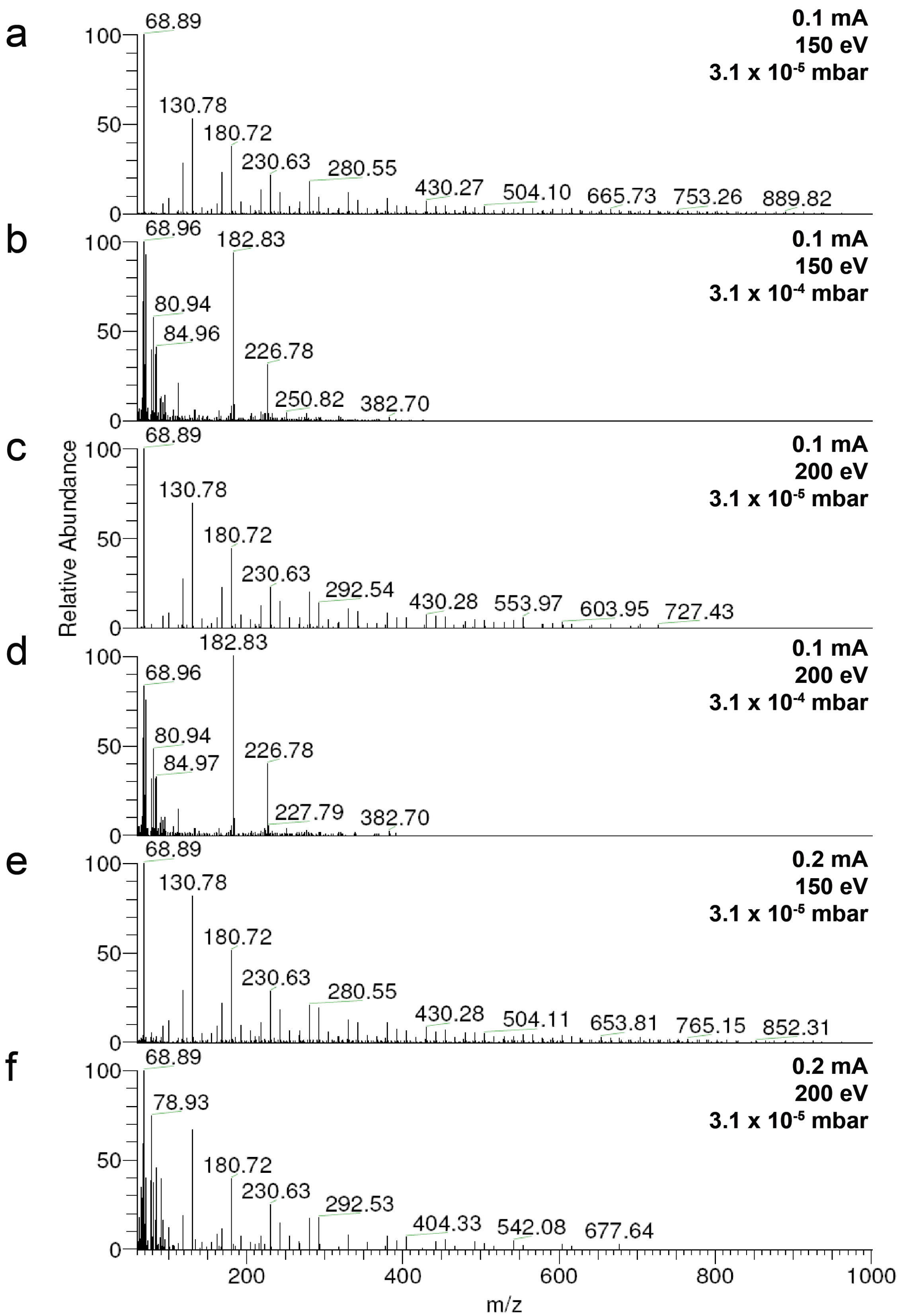

Supplementary Figure 2 – LR CI Mass Spectra of PFK in Isobutane at 200^{0}C and different conditions. Low resolution mass spectra of PFK acquired on Thermo Finnigan MAT 95 XP double focusing sector field spectrometer operated in isobutane reactant gas at 150^{0}C while varying (a-f) current filament of (0.1-0.2 mA), electrons energy source (150-200 eV) and reagent gas pressure (3.1 x 10^{-5} - 3.1 x 10^{-4} mbar).

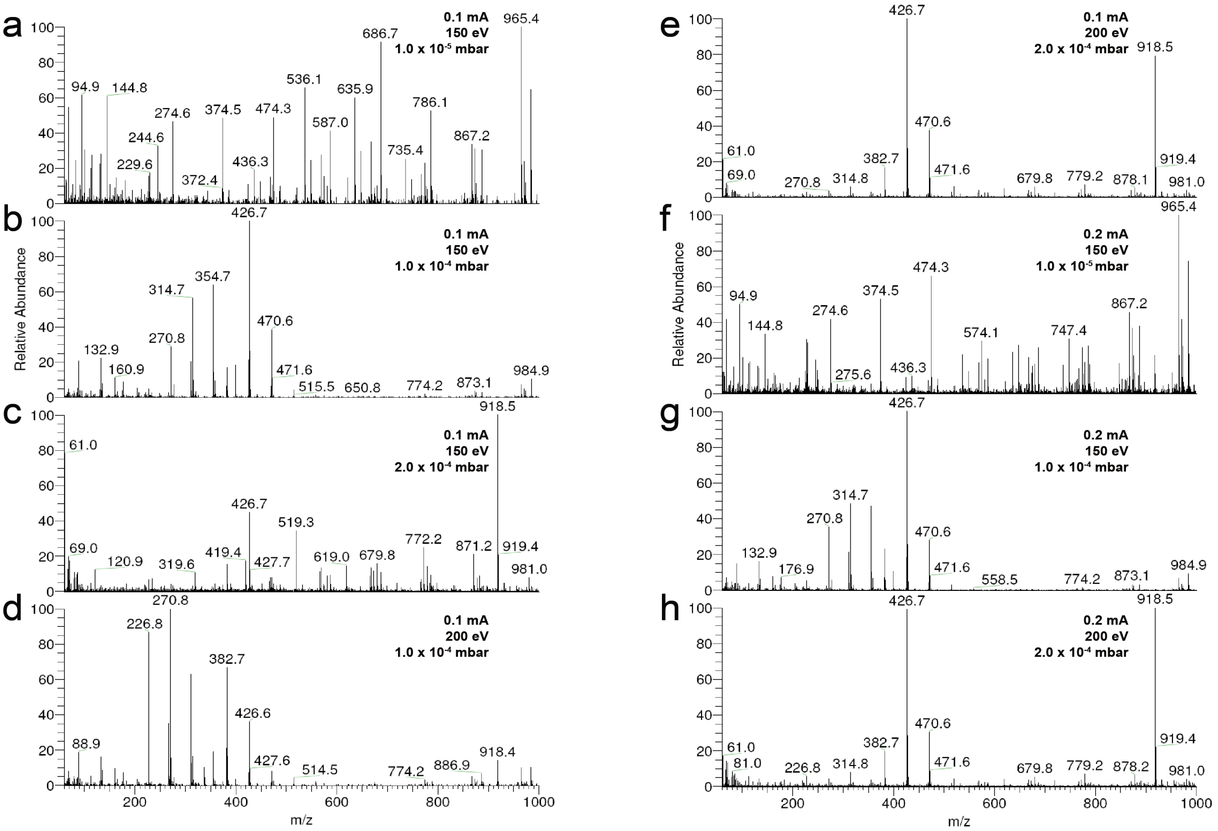

Supplementary Figure 3 – LR CI Mass Spectra of Ultramark 1621 in Isobutane at 150^{0}C and different conditions. Low resolution mass spectra of Ultramark 1621 acquired on Thermo Finnigan MAT 95 XP double focusing sector field spectrometer operated in isobutane reactant gas at 150^{0}C while varying (a-h) current filament of (0.1-0.2 mA), electrons energy source (150-200 eV) and reagent gas pressure (1.0 x 10^{-5} - 2.0 x 10^{-4} mbar).

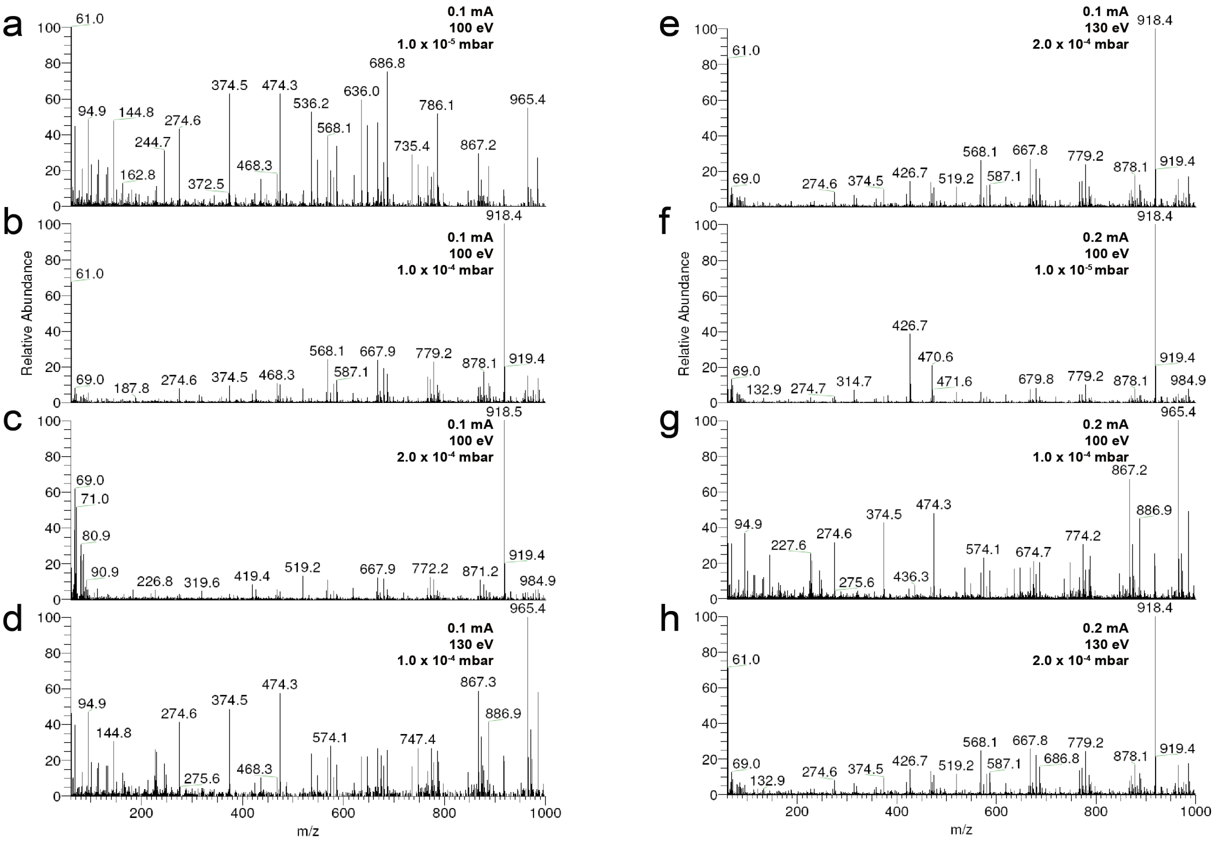

Supplementary Figure 4 – LR CI Mass Spectra of Ultramark 1621 in Isobutane at 200^{0}C and different conditions. Low resolution mass spectra of Ultramark 1621 acquired on Thermo Finnigan MAT 95 XP double focusing sector field spectrometer operated in isobutane reactant gas at 200^{0}C while varying (a-h) current filament of (0.1-0.2 mA), electrons energy source (100-130 eV) and reagent gas pressure (1.0 x 10^{-5} - 2.0 x 10^{-4} mbar).

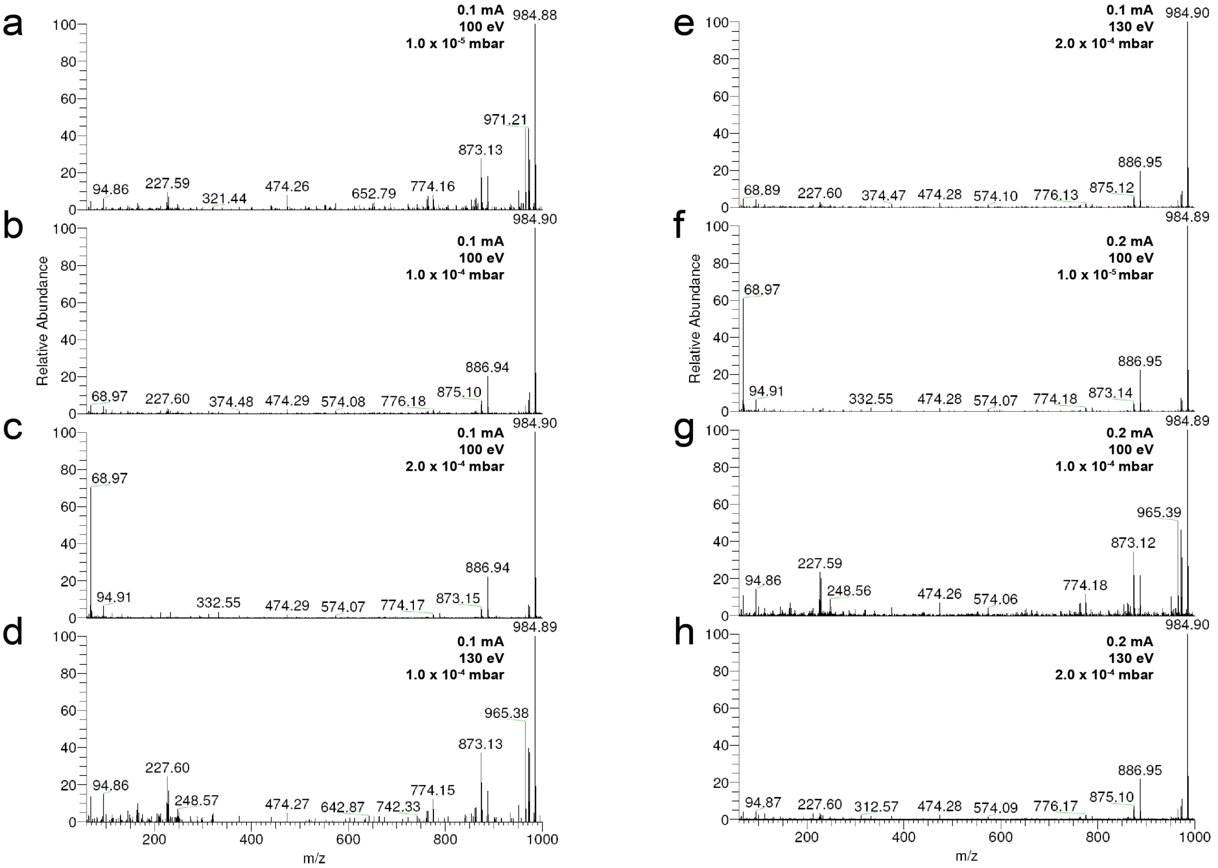

Supplementary Figure 5 – LR CI Mass Spectra of Ultramark 1621 in Methane at 200^{0}C and different conditions. Low resolution mass spectra of Ultramark 1621 acquired on Thermo Finnigan MAT 95 XP double focusing sector field spectrometer operated in isobutane reactant gas at 200^{0}C while varying (a-h) current filament of (0.1-0.2 mA), electrons energy source (100-130 eV) and reagent gas pressure (1.0 x 10^{-5} - 2.0 x 10^{-4} mbar).

Supplementary Tables

Supplementary Table 1. Optimization Conditions for PFK with Isobutane carrier gas.

	PFK with Isobutane at 200^{0}C					
	**1**	**2**	**3**	**4**	**5**	**6**
**Filament Current (in mA)**	0.1	0.1	0.1	0.1	0.2	0.2
**Electron Energy (in eV)**	150	150	200	200	150	200
**Pressure (in mbar)**	3.1×10^{-5}	3.1×10^{-4}	3.1×10^{-5}	3.1×10^{-4}	3.1×10^{-5}	3.1×10^{-5}
**Average Count (in bin)**	22	14	9	15	30	6
**Average RSD**	183.77	144.02	134.43	183.33	188.08	101.19
**Average - Median**	1.01	1.58	1.01	1.58	1.01	1.58

Supplementary Table 2. Optimization Conditions for Ultramark 1621 with Isobutane and Methane carrier gases.

a

	Ultramark 1621 with Isobutane at 150^{0}C							
	**1**	**2**	**3**	**4**	**5**	**6**	**7**	**8**
**Intensity (a.u.)**	62,000	100,000	1,600,000	80,000	1,600,000	295,000	865,000	2,300,000
**Filament Current (in mA)**	0.1	0.1	0.1	0.1	0.1	0.2	0.2	0.2
**Electron Energy (in eV)**	150	150	150	200	200	150	150	200
**Pressure (in mbar)**	1.0×10^{-5}	1.0×10^{-4}	2.0×10^{-4}	1.0×10^{-4}	2.0×10^{-4}	1.0×10^{-5}	1.0×10^{-4}	2.0×10^{-4}
**Average Count (in bin)**	44	38	44	27	44	44	43	44
**Average RSD**	188.42	201.37	169.35	203.21	184.34	155.81	197.73	170.90
**Average - Median**	2.26	0.87	0.78	1.10	0.59	1.85	0.85	0.68

b

	Ultramark 1621 with Isobutane at 200^{0}C							
	**1**	**2**	**3**	**4**	**5**	**6**	**7**	**8**
**Intensity (a.u.)**	170,000	537,000	598,000	39,000	133,000	322,000	233,000	1,259,000
**Filament Current (in mA)**	0.1	0.1	0.1	0.1	0.1	0.1	0.2	0.2
**Electron Energy (in eV)**	150	150	150	200	200	200	150	150
**Pressure (in mbar)**	1.0×10^{-5}	1.0×10^{-4}	2.0×10^{-4}	1.0×10^{-5}	1.0×10^{-4}	2.0×10^{-4}	1.0×10^{-4}	2.0×10^{-4}
**Average Count (in bin)**	44	45	44	42	45	45	45	45
**Average RSD**	188.50	154.35	149.19	169.36	155.85	161.40	160.34	148.09
**Average - Median**	2.22	0.87	0.50	1.85	0.77	0.50	1.49	0.93

c

	Ultramark 1621 with Methane at 200^{0}C							
	**1**	**2**	**3**	**4**	**5**	**6**	**7**	**8**
**Intensity (a.u.)**	63,000	540,000	750,000	26,000	257,000	360,000	193,000	1,500,000
**Filament Current (in mA)**	0.1	0.1	0.1	0.1	0.1	0.1	0.2	0.2
**Electron Energy (in eV)**	100	100	100	130	130	130	100	100
**Pressure (in mbar)**	1.0×10^{-5}	1.0×10^{-4}	2.0×10^{-4}	1.0×10^{-5}	1.0×10^{-4}	2.0×10^{-4}	1.0×10^{-5}	1.0×10^{-4}
**Average Count (in bin)**	29	43	42	44	45	44	44	45
**Average RSD**	135.46	144.54	178.06	134.04	141.60	166.27	132.88	140.54
**Average - Median**	0.91	0.28	0.35	0.84	0.28	0.39	0.72	0.31

1. Brenton, A. G.; Godfrey, A. R., Accurate Mass Measurement: Terminology and Treatment of Data. *Journal of the American Society for Mass Spectrometry* **2010,** *21* (11), 1821-1835.
2. Jing, L.; Li, C.; Wong, R. L.; Kaplan, D. A.; Amster, I. J., Improved mass accuracy for higher mass peptides by using SWIFT excitation for MALDI-FTICR mass spectrometry. *J Am Soc Mass Spectrom* **2008,** *19* (1), 76-81.
3. Pretorius, E., Influence of acceleration voltage on scanning electron microscopy of human blood platelets. *Microscopy research and technique* **2010,** *73* (3), 225-8.
